# High Sensitivity Terahertz Biosensor Based on Mode Coupling of a Graphene/Bragg Reflector Hybrid Structure

**DOI:** 10.3390/bios11100377

**Published:** 2021-10-08

**Authors:** Yamei Liu, Qiwen Zheng, Hongxia Yuan, Shenping Wang, Keqiang Yin, Xiaoyu Dai, Xiao Zou, Leyong Jiang

**Affiliations:** 1 School of Physics and Electronics, Hunan Normal University, Changsha 410081, China; yamei_cl@163.com (Y.L.); qiwen@hunnu.edu.cn (Q.Z.); yuanhx@hunnu.edu.cn (H.Y.); shenpingwang@smail.hunnu.edu.cn (S.W.); keqiangYin@hunnu.edu.cn (K.Y.); 2 College of Electrical and Information Engineering, Hunan University, Changsha 410082, China; xiaoyudai@126.com

**Keywords:** terahertz biosensor, Tamm plasmons, mode coupling, graphene

## Abstract

In this work, a high-sensitivity terahertz (THz) biosensor is achieved by using a graphene/Bragg reflector hybrid structure. This high-sensitivity THz biosensor is developed from the sharp Fano resonance transmission peak created by coupling the graphene Tamm plasmons (GTPs) mode to a defect mode. It is found that the proposed THz biosensor is highly sensitive to the Fermi energy of graphene, as well as the thickness and refractive index of the sensing medium. Through specific parameter settings, the composite structure can achieve both a liquid biosensor and a gas biosensor. For the liquid biosensor, the maximum sensitivity of > 1000 °/RIU is obtained by selecting appropriate parameters. We believe the proposed layered hybrid structure has the potential to fabricate graphene-based high-sensitivity biosensors.

## 1. Introduction

A optical biosensor detects biological information through optical signals, and it can convert invisible biological phenomena or measured biological signals into optical signals that are easy to quantify and measure [1]. It not only provides non-contact, label-free and non-destructive measurements, but also presents less interference and high sensitivity [2,3]. These characteristics make optical biosensors very popular in the detection of heavy metal ions [4], pathogenic microbes detection [5], drug testing [6], detection of biological small molecules [7] and other fields. In recent years, with the development of micro-nano processing technology, optical biosensors based on micro-nano structures have become the main research direction due to their small size and easy integration. The realization of optical biosensors based on photonic crystals [8], carbon nanotubes [9], microring cavity structure [10], toroidal dipole resonance [11], THz plasmonic [12] and other micro-nano structures and mechanisms has been reported. In particular, surface plasmon resonance (SPR) is widely used in the field of micro-nano optical sensing technology because its resonance peak is very sensitive to small changes in external characteristics. Schemes combining SPR technology with various materials and structures have been proposed one after another [13,14,15]. Additionally, in order to obtain highly sensitive, simple-structured, and dynamically controllable optical biosensor schemes, researchers have been trying to develop new optical biosensor schemes based on new materials and mechanisms [16,17,18,19]. In recent years, many researchers have been attracted by two-dimensional materials for their unique and excellent photoelectric properties, among which graphene is a typical representative. Graphene exhibits certain metallic properties under certain conditions, so it can be used to stimulate some strange electromagnetic responses [20]. Furthermore, the dielectric constant and electrical conductivity of graphene are dynamically controllable. These characteristics offer a unique advantage in realizing enhanced biosensors [21,22,23]. For example, Wu et al. proposed a graphene-based SPR biosensor, the sensitivity of which increased by nearly 25% in comparison with traditional gold thin-film SPR biosensors [24]; Sreekanth et al. developed a novel graphene-based biosensor from theoretical research. They cross-superimposed graphene onto polymethyl methacrylate to form a one-dimensional photonic crystal and then used prism coupling technology to obtain a stable biosensor with high phase sensitivity [25]; based on traditional SPR biosensors, Zeng et al. introduced graphene and molybdenum disulfide to design a new type of reliable and excellent SPR biosensor [26]. The successful application of graphene in biosensors lays the foundation for the application of two-dimensional materials in biosensors. Naturally, the application of other two-dimensional materials such as black phosphorus and transition metal dichalcogenides in biosensors has also become possible. The prospects of optical biosensors are very bright, and optical biosensors based on two-dimensional materials will be a research focus in optical biosensing and an inevitable trend corresponding to the development of materials science and information science.

In recent years, Tamm plasmons (TPs), a type of surface wave confined on the contact surface of two different media which are easily excited and have strong locality to light, have attracted a lot of interest from researchers [27]. Compared with SPR, TPs can be excited by TM-polarized and TE-polarized waves [28] and can also excite photonic crystals without meeting specific incident angles. Moreover, TPs have stronger localization [29] and are more sensitive to the changes of the boundary environment. Therefore, biosensors based on TPs have naturally attracted the attention of researchers. Sensors based on TPs have been designed for blood component detection [30] and temperature sensing [31]. In addition, Tang et al. realized a high-sensitivity optical biosensor in a terahertz band by using a graphene/Bragg reflector composite structure [32]; Ruan et al. realized an ultra-sensitive terahertz biosensor by coupling graphene surface plasmons to planar waveguide modes [33]; and recently, Li et al. proposed a novel magneto-optical TPs sensor with high sensitivity for fluid detection [34].

In this paper, we theoretically propose a multilayer hybrid structure composed of graphene and Bragg reflector. In this hybrid structure, a highly sensitive terahertz biosensor is achieved by coupling the TPs mode excited by the graphene and Bragg reflector with the defect mode of the photonic crystal to form Fano Resonance. We found that parameters such as the Fermi energy of graphene and the thickness and refractive index of the sensing layer have significant effects on the sensitivity of the whole structure. At the same time, the sensing scheme can also be used for gas sensing through structural parameter adjustment. Meanwhile, through parameter optimization, the refractive index sensitivity of the whole structure can reach a level of more than 1000 °/RIU. The scheme is simple in structure, easy to adjust, and highly sensitive to tiny changes. We believe it has potential applications in the field of biosensors.

## 2. Materials and Methods

**Figure 1:**
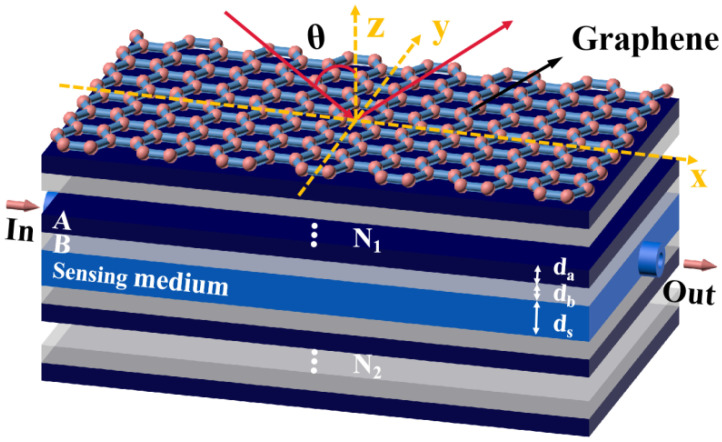
Schematic diagram of a terahertz biosensor based on graphene/Bragg reflector composite structure.

We consider a hybrid structure based on the graphene/Bragg reflector, which is composed of graphene, photonic crystal 1, a sensing layer, and photonic crystal 2 from top to bottom. For simplicity, we assume that the background material at the upper and lower ends of the structure is air. It should be noted that both the addition and removal of the substrate has little effect on the sensing performance according to the calculation results. Therefore, we do not consider the case of adding the substrate for the time being in order to simplify the calculation. Meanwhile, considering the practical application scenario of biosensors, we set the sensing liquid/gas inlet and outlet in the sensing medium layer of the structure, as shown in Figure 1. In the actual device preparation, we can add two baffles on both sides of the sensing layer. These two baffles do not affect the calculation and experimental results in the structure, but only play a supporting role. We assume that the incident light propagates in the air and hits the graphene at θ. The graphene is placed on the top of the structure. Photonic crystal 1, a Bragg mirror structure with a period of $N_{1}$, is composed of two different dielectrics, A and B, whose refractive indexes and thicknesses are, respectively, expressed as $n_{a},d_{a},n_{b},\;\mathrm{and}\;d_{b}$. In fact, in order to facilitate the excitation of TPs, it is necessary to add an isolation layer between the graphene and photonic crystal 1. However, since photonic crystal 1 itself is composed of a dielectric stack, in the case of a relatively large period, the uppermost dielectric layer of the photonic crystal can also approximate to an isolation layer, so there is no need to add an isolation layer. Photonic crystal 2 with a period of $N_{2}$ is similar in composition to photonic crystal 1, but it has a symmetrical distribution with the dielectric layer of photonic crystal 1; the refractive index and thickness are $n_{s}$ and ds_,_ respectively. The sensing layer is placed between the two photonic crystals. Such a structure allows defect modes to appear in photonic crystals.

We selected the terahertz band and set the center wavelength to $\lambda_{c}=300\;\mu m$. In the following calculations, we set the period of the photonic crystal to $N_{1}{=N}_{2}=12$. The refractive indexes of dielectrics A and B were assumed to be non-dispersive and were, respectively, set as: $n_{a}=2.35$ and $n_{b}=1.37$. This can be realized by glass and ${MgF}_{2}$ in actual optical materials [35,36]. In addition, the thicknesses of dielectrics A and B were set to $d_{a}=31.1\;\mu m$ and $d_{b}=44.9\;\mu m$_,_ respectively, for a better coupling effect. The absorption of the sensing layer has a certain impact on the sensing sensitivity in the case of terahertz band biosensors, but for simplicity we ignore the influence of absorption on the sensing performance. When the structure was used for liquid sensing, we set the refractive index and thickness of the sensing layer to $n_{s}=1.33$ and $d_{s}=487\;\mu m$_,_ respectively. As for the graphene on top of photonic crystal 1, which is only one atom thick (namely, 0.34 nm), its photoelectric properties are expressed by conductivity. Within the local random phase approximation and terahertz frequency range, the intra-conductivity of graphene is much greater than inter-conductivity. Therefore, the conductivity of monolayer graphene can be approximately expressed as [37]:

(1) σ ≈ ie2EFπћ2(ω+iτ)’,

where ћ is the simplified Planck constant and ω is the angular frequency of the incident beam; e and τ represent the elementary electric charge and the relaxation time, respectively. $E_{F}$ is the Fermi energy which is closely related to the carrier density ($n_{2D}$), and $E_{F}{=\text{ћ}V}_{F}\sqrt{{2\pi n}_{2D}}$; $V_{F}\;\approx \;\;10^{6}\;m/s$ represents the Fermi velocity of the electron. It provides a way for the gate voltage to flexibly and dynamically manipulate the conductivity characteristics of graphene. In the following calculations, the Fermi energy and the relaxation time of graphene are taken as $E_{F}=0.95\;\mathrm{eV}$ and τ= 1 ps_,_ respectively. In the calculation we found that the sensing performance of the multilayer graphene structure is not significantly improved, so we only considered the case of single-layer graphene.

In order to obtain the sensing performance of the whole structure, we need to calculate the reflectivity of the structure by the classical and mature transfer matrix method [38]. For simplicity, this paper only considers the case of TM polarization. In this case, the transfer matrix between graphene and dielectric can be expressed as [38]:

(2) $$D_{i\to A}=\frac{1}{2}\left[\begin{array}{c} {1+\eta }_{iA}{+\xi }_{iA}\\ {1-\eta }_{iA}{+\xi }_{iA} \end{array}\begin{array}{c} {1-\eta }_{iA}{-\xi }_{iA}\\ {1+\eta }_{iA}{-\xi }_{iA} \end{array}\right],$$

where $\eta_{iA}={\;\varepsilon }_{i}k_{Az}/\varepsilon_{A}k_{iz}$ and $\xi_{iA}={\sigma k}_{Az}/\varepsilon_{0}\varepsilon_{A}\omega$; $k_{iz}$ and $k_{Az}$ are the wave vector components of electromagnetic waves propagating in the air layer and dielectric A, respectively. In the calculation model we used, it can be found from expression (2) that since graphene is only 0.34 nm thick, its conductivity characteristics can be reflected by boundary conditions and it is no longer regarded as a single layer. Combined with the propagation matrix of electromagnetic waves in each dielectric layer, the transmission matrix of the entire system can be expressed as [38]:

(3) $${M=D}_{i\to A}{\left(p_{A}D_{A\to B}p_{B}D_{B\to A}\right)}^{N_{1}-1}p_{A}D_{A\to B}p_{B}D_{S\to B}{\left(p_{B}D_{B\to A}p_{A}D_{A\to B}\right)}^{N_{2}-1}p_{B}D_{B\to A}p_{A}D_{A\to o},$$

according to which the reflection coefficient of the whole structure can be obtained by $r=\;M_{21}/M_{11}$. Thus, the reflectivity $R={\;|r|}^{2}$ is obtained.

As we know, sensitivity is the core index of a biosensor’s performance. In this paper, we define the sensitivity of the structure as [39]:

(4) $$S=\frac{\Delta \theta }{{\Delta n}_{S}},$$

where ${\Delta n}_{S}$ represents the change of refractive index in the sensing medium layer, and Δθ represents the angle change of Fano resonance peak.

## 3. Results and Discussion

**Figure 2:**
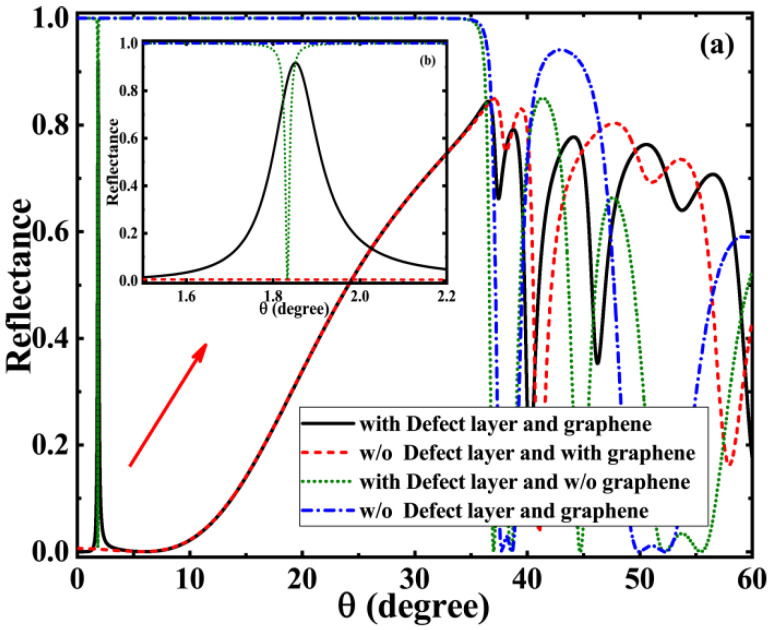
Application of the structure as a liquid biosensor: (**a**) the curve of reflectance with incident angle when the structure is loaded with defect layer and graphene (black solid line), unloaded with defect layer but loaded with graphene (red short dash line), loaded with defect layer but unloaded with graphene (green short dot line), and unloaded with defect layer and graphene (blue short dash dot line); (**b**) partial enlargement of reflectance curve.

We know that both classical SPR biosensors and other biosensors such as Bloch surface wave biosensors could perceive the slight change in the characteristics of the sensing layer medium (e.g., refractive index) by observing the change of the reflection peak. Our work is no exception, so we first focus on the variation of the reflectance of the proposed structure with the incident angle. Additionally, in order to facilitate a comparison and highlight the role of graphene and defect layer, we put together the reflectance curves of several combinations based on the parameters set in the previous section, as shown in Figure 2. We found that if photonic crystals 1 and 2 are simply put together, there is nothing special about the whole structure. The reflectance is reflected as a typical photonic band gap in the range of 0° to 35°, which is clearly demonstrated by the blue short dash dot line in the figure. The electromagnetic wave in this case cannot penetrate through the whole structure, let alone the sensing detection. Placing a sensing layer in the middle of a symmetric photonic crystal is equivalent to the introduction of a defect. In this case, we find an obvious sharp reflection peak near 1.8°, which is a typical defect mode feature (thus, in the manuscript we also refer to the sensing layer as the defect layer). In addition, if we add a layer of graphene on one side of the two-photon crystal alone, we can also observe a clear frequency-dependent reflection peak. In the case of a fixed frequency, the change of reflection with angle is characterized by a relatively wide reflection peak, which is the typical excitation of graphene Tamm plasmons (GTPs). Furthermore, if the graphene and the defect layer are loaded simultaneously, mode coupling would occur under suitable parameter modulation. We find that the GTPs reflection peak and the defect mode reflection peak overlap each other to produce a sharp upward reflection peak, which is a typical Fano resonance phenomenon. Since this sharp and upward reflecting peak is sensitive to both the GTPs mode and the defect mode, small changes in the sensing layer have a significant effect on the Fano resonance, which provides conditions for high-sensitivity sensing detection.

**Figure 3:**
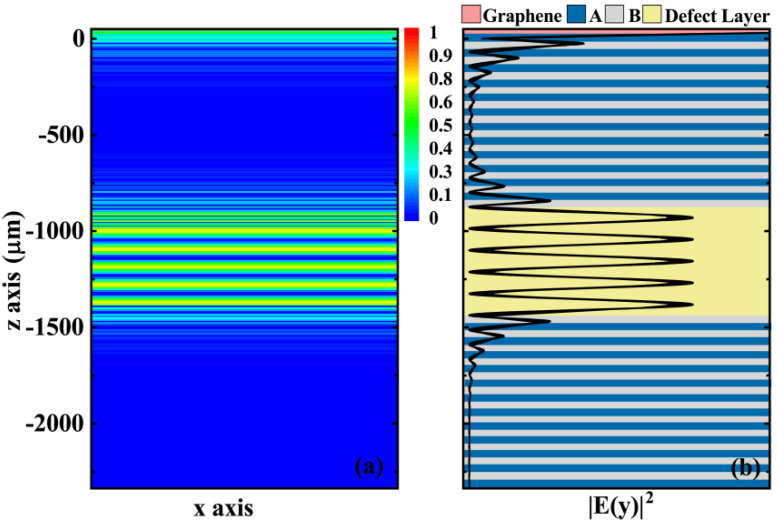
Field distribution of the graphene/Bragg reflector hybrid structure: (**a**) colorful diagram of the structure; (**b**) one-dimensional electric field distribution curve of the structure (all parameters are consistent with those in Figure 2).

In order to further show the generation of the GTPs mode and defect mode in the proposed structure, we plot the normalized electric field distributions of the whole structure at incidence of center wavelength based on the structure and material parameters in Figure 2, and the results are shown in Figure 3. Figure 3 shows the field distribution of the structure at different positions in the forms of a normalized colorful diagram and a curved plot, respectively. In order to better understand the field distribution in different media, we set the position of graphene to 0 and reflected different parts of the structure as different background colors in Figure 3b according to a specific scale. From Figure 3, we can see that the electric field shows a clear field enhancement effect near the graphene and at the defect layer (or the sensing layer). The field enhancement near graphene indicates the generation of the GTPs mode, which is closely related to the local field enhancement of TPs. The field enhancement of the sensing layer indicates the generation of defect modes, which is also related to the local field enhancement of defect modes. The field distribution of Figure 3 offers effective support for the reflectance curves in Figure 2 and predicts the occurrence of mode coupling.

**Figure 4:**
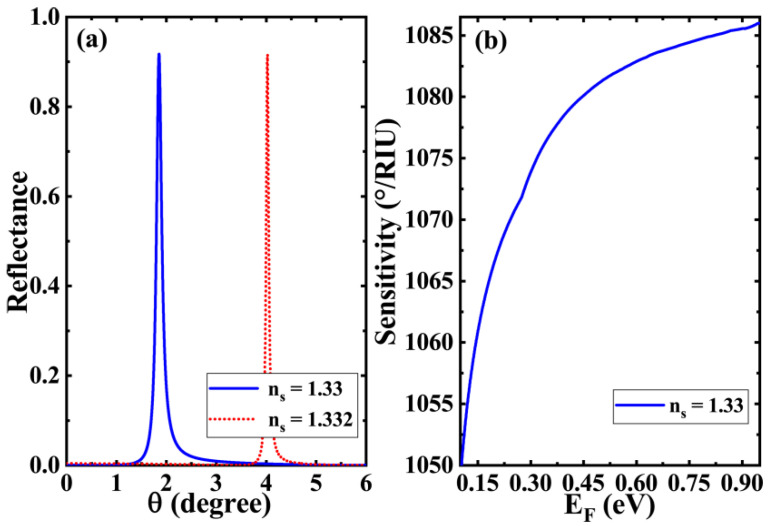
(**a**) Reflectance of the biosensor structure with respect to the refractive index of different sensing layers at $E_{F}=0.95\;\mathrm{eV}$; (**b**) sensitivity curve of the biosensor structure relative to the Fermi energy.

Next, we discuss the sensitivity of the whole sensor structure. For the sensing layer containing biomolecules, we chose an aqueous solution with an initial refractive index of $n_{s}=1.33$. We assumed that the change of refractive index of the sensing layer is ${\Delta n}_{s}=0.002$ due to the interaction of biomolecules. This refractive index change is smaller than the values taken in our previous work. It can be found from Figure 4a that when the refractive index of the aqueous solution of the sensing layer is $n_{s}=1.33$, a sharp Fano resonance peak appears at 1.8°. This is the same as in Figure 2. These sharp resonance peaks mean that even small changes in structural parameters can cause significant shifts in the position of Fano resonance peaks. When the refractive index changes to $n_{s}=1.332$ in aqueous solution due to subtle changes in biological characteristics, the Fano resonance peak of the structure would shift to a higher angle by more than 2°, and the sensitivity reflected in this process would exceed 1085 °/RIU. This means the Fano resonance peak based on mode coupling has a great impact on the structure of material parameters, which is particularly useful in high-sensitivity biosensors. It is worth mentioning that the TPs can also be excited under TE polarization [28]. However, we find that the sensing characteristics of the structure under TE polarization highly resembles those under TM polarization. Hence, we only briefly mention it in the discussion part. We can also obtain the sensing characteristics of the structure under TE polarization by similar calculation methods [38]. Additionally, we further calculate the figure of merit (FOM) in this case. The calculation expression of FOM is given in reference [32]. It can be seen from the FOM definition expression that the FOM of this upward reflection peak will be very high. Based on the parameters shown in Figure 4a, the FOM value of 8482 RIU^−1^ is obtained through calculation, which is at a high level among similar biosensors. The variation pattern of this biosensor’s sensitivity in relation to the Fermi energy of graphene is illustrated in Figure 4b. The Fermi energy of graphene takes a certain value in the abscissa; the corresponding sensitivity when the refractive index of the sensing layer changes from 1.33 to 1.332 is taken as the ordinate. It is found that even a low Fermi energy can make the sensitivity of the structure above 1000 °/RIU. At the same time, the increase in the Fermi energy of graphene leads to a significant enhancement in the sensitivity of the structure. However, it should be noted that for biosensors, although higher sensitivity means a better performance, combined with the curve trend in Figure 4b, the sensitivity of the structure does not necessarily rise continuously with the increase in Fermi energy. On the one hand, the Fermi energy of graphene cannot increase continuously, which is determined by the structural properties of graphene materials. In general, the values of Fermi energy of graphene are below 1 eV [40]. On the other hand, even if the Fermi energy is taken to be larger than 1 eV in a theoretical calculation, the mode coupling would be weakened or even disappear because the GTPs mode condition is not satisfied. Therefore, in the selection of initial parameters, we chose the Fermi energy of graphene as $E_{F}=0.95\;\mathrm{eV}$. It should also be noted that the Fermi energy of 0.95 eV can be achieved theoretically, but it is already very high [40]. Therefore, in practical applications, we generally set Fermi energy to a smaller value. The proposed structure is also compared with those in other reported papers and it is found that our structure has better sensitivity, better FOM and simple structure, as shown in Table 1.

**Figure 5:**
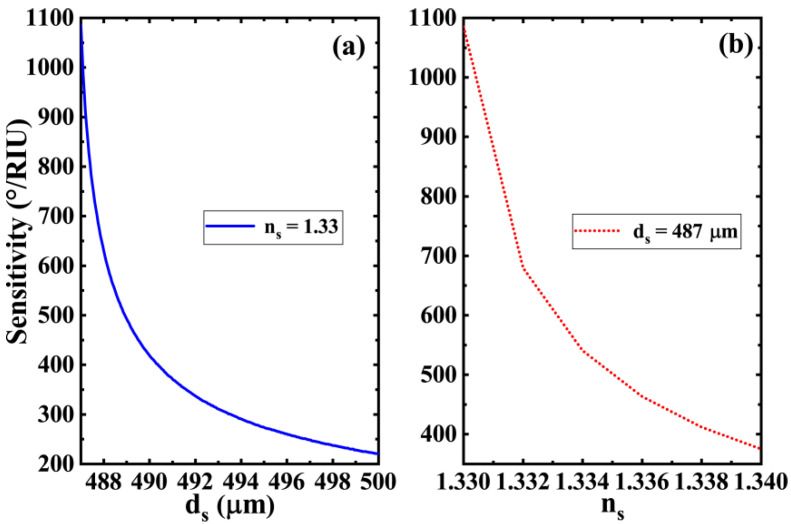
Effects of (**a**) the thickness and (**b**) the refractive index of the sensing layer on the sensitivity of biosensor (other parameters are the same as those in Figure 2).

We know that the position of the defect mode peak of photonic crystals is greatly affected by the defect structure. In the scheme of this paper, we place the sensing layer at the position of the defect layer. It is conceivable that the structural parameters of the sensing layer itself have a significant impact on the sensitivity characteristics of the whole sensor structure. Therefore, it is very necessary to investigate the relationship between the structural parameters of the sensing layer and the sensitivity of the sensor to improve its performance. The variations of the sensitivity as a function of the thickness and refractive index of the sensing layer are shown in Figure 5. In Figure 5a, there is a clear monotonic decreasing relationship between the sensitivity of the biosensor and the thickness of the sensing layer. This relationship predicts that if we want to further improve the sensitivity of the sensor, it would be a good choice to set the thickness of the sensing layer to a smaller value. However, this does not mean that the smaller the thickness of the sensing layer, the better the performance. On the one hand, a thinner sensing layer requires higher manufacturing precision and a superior technique; on the other hand, because the thickness of the sensing layer is closely related to the position of the GTPs mode and defect mode, the decreasing thickness of the sensing layer also makes the Fano resonance peak disappear gradually, and leads to the weakening of the sensing characteristics of the whole structure. Therefore, in the curve we draw, the thicknesses of the sensing layer are above $d_{s}=487\;\mu m$. In addition, the increase in the refractive index of the sensing layer also makes the sensitivity of the biosensor gradually decrease, as shown in Figure 5b. The refractive indexes of aqueous solution in biosensing normally change in a very small range, most of which increase slightly based on n_s_ = 1.33. Therefore, biological detection in aqueous solutions with a refractive index of about 1.33 generally exhibits high sensitivity.

**Figure 6:**
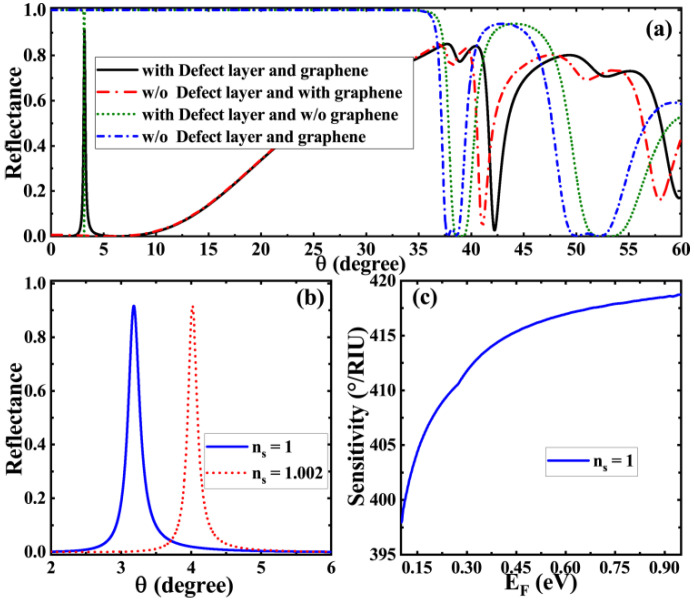
Application of the structure as a gas biosensor: (**a**) the curve of reflectance with incident angle when the structure is loaded with defect layer and graphene (black solid line), unloaded with defect layer and loaded with graphene (red short dash line), loaded with defect layer and unloaded with graphene (green short dot line), and unloaded with defect layer and graphene (blue short dash dot line); (**b**) the curve of reflectance with refractive index in relation to the sensing layer at $E_{F}=0.95\;\mathrm{eV}$; (**c**) the variation curve of sensitivity with respect to Fermi energy.

In the previous discussion, we assumed that the sensing layer is an aqueous solution, so the structure in Figure 1 can realize the biosensing of an aqueous solution through an appropriate parameter selection. Can this structure be used for gas sensing if its sensing layer is filled with gas? To answer this question, based on the structure and material parameters of liquid biosensors, we adjusted the thickness of the sensing layer to $d_{s}=60\;\mu m$ and the refractive index to $n_{s}=1$ [46]; other parameters remained unchanged. The obtained variation curve of reflectivity with incident angle under different conditions is shown in Figure 6a. It can be seen from the curve that when the medium of the sensing layer is gas, the asymmetric Fano resonance peak corresponding to the coupling of GTPs would appear and the defect mode would also occur. The resonance peak is also very sensitive to the slight change of the refractive index of the sensing layer. As can be seen in Figure 6b, even a small change of 0.002 in the refractive index of the gas leads to a significant shift in the asymmetric Fano resonance peak. In addition, the increase in graphene Fermi energy also monotonically improves the sensitivity of the gas sensor, as shown in Figure 6c, which is the reason that we chose a higher Fermi energy.

## 4. Conclusions

To summarize, we propose a highly sensitive terahertz biosensor based on a graphene/Bragg reflector composite structure. In this composite structure, sharp Fano resonance is achieved by the coupling of the TPs mode (generated by the graphene and Bragg reflector) and the defect mode (generated by the symmetric Bragg reflector). This sharp Fano resonance creates conditions for the realization of a high-sensitivity refractive index sensor. Through parameter optimization, the structure enables biosensing measurements for not only solutions, but also gases. Taking the liquid sensor as an example, we found that the structure can achieve a refractive index sensitivity of greater than 1000 °/RIU by optimizing the structure and graphene parameters. Meanwhile, the sensitivity of the structure is highly related to the thickness and refractive index of the sensing medium as well as the Fermi energy of graphene. The proposed sensing scheme is highly sensitive and simple in structure with low processing requirements. Therefore, it is expected to find potential applications in the field of biosensing based on micro-nano structures.

## Figures and Tables

**Table 1:** Comparison between different refractive index sensing methods.

Ref.	Mechanism	Structure	Sensitivity	FOM (RIU^−1^)	Frequency Range
[32]	OTSs sensor	Graphene-Bragg reflector structure	400 º/RIU	60	THz
[33]	Mode coupling sensor	Otto structure	3260 RIU^−1^	/	THz
[41]	OTSs sensor	Bragg reflector-Graphene structure	517.9 º/RIU	222.9	THz
[42]	Bloch surface wave sensor	Prism-photonic crystal structure	117 º/RIU	283	THz
[43]	SPR sensor	Grating structure	237 º/RIU	95	Near Infrared
[44]	SPR sensor	Otto structure	34.11 º/RIU	1150	THz
[45]	Defect-mode coupling sensor	Bragg reflector structure (with defect layer)	810 nm/RIU	9679	Near Infrared
This work	Mode coupling sensor	Graphene-Bragg reflector structure (with defect layer)	1085 º/RIU	8482	THz
